# Mushrooms of the Genus *Ganoderma* Used to Treat Diabetes and Insulin Resistance

**DOI:** 10.3390/molecules24224075

**Published:** 2019-11-11

**Authors:** Katarzyna Wińska, Wanda Mączka, Klaudia Gabryelska, Małgorzata Grabarczyk

**Affiliations:** Department of Chemistry, Wroclaw University of Environmental and Life Sciences, Norwida 25, 50-375 Wroclaw, Poland; klaudia.gabryelska@gmail.com

**Keywords:** *Ganoderma*, antidiabetic, polysaccharides, triterpenoids, meroterpenoids

## Abstract

Pharmacotherapy using natural substances can be currently regarded as a very promising future alternative to conventional therapy of diabetes mellitus, especially in the case of chronic disease when the body is no longer able to produce adequate insulin or when it cannot use the produced insulin effectively. This minireview summarizes the perspectives, recent advances, and major challenges of medicinal mushrooms from *Ganoderma* genus with reference to their antidiabetic activity. The most active ingredients of those mushrooms are polysaccharides and triterpenoids. We hope this review can offer some theoretical basis and inspiration for the mechanism study of the bioactivity of those compounds.

## 1. Introduction

Diabetes is a metabolic disorder caused by a lack of insulin and insulin dysfunction characterized by hyperglycemia. Nowadays, diabetes has become a serious problem. This disease is no longer considered congenital and rare.

It affects more and more people and is the subject of many discussions. The International Diabetes Federation in the eighth edition of Global Diabetes Map published data according to which in 2017 the number of adults of diabetes worldwide patients (20–79 years) reached 415 million. It should be noted that in 2000 there were “only” 151 million such patients. Estimates suggest that by 2045 the number of diabetic patients could reach 629 million [1].

The previous classification of diabetes has distinguished two types of this disease. Differentiation of these groups was mainly based on the presence (type 1) or absence (type 2) of autoantibodies against pancreatic islet β-cell antigens. The next criterion was the age at which the patient was diagnosed. There was also a third subgroup containing cases of adult patients with latent autoimmune diabetes (LATA). This type of diabetes was defined by the presence of glutamic acid decarboxylase antibodies (GADA). According to the latest research presented by Ahlqvist et al. in 2018 [2], this disease includes five types. The first type was described as severe autoimmune diabetes (SAID). Diabetes of type 1 is usually found in young people with relatively low BMI. It is characterized by poor metabolic control, insulin deficiency, and presence of GADA. The second type is also juvenile diabetes but is GADA negative. This type is characterized, like type 1, by relatively low BMI, low insulin secretion (low HOMA2-B index), and poor metabolic control. Type 2 diabetes is defined as severe insulin-deficient diabetes (SIDD). The third type of diabetes is not age-related but is associated with obesity (high BMI). This type is characterized by insulin resistance (high HOMA2-IR index). It has been named as severe insulin resistant diabetes (SIRD). The fourth type of diabetes is also associated with obesity but not by insulin resistance. This type is defined as mild obesity-related diabetes (MOD). The fifth type of diabetes occurs in the elderly and is milder (minor metabolic changes are observed). Patients with this type of disease can be controlled with medication without need of insulin injection. This type is defined as mild age-related diabetes (MARD). This division allows a better adaptation of the treatment method to the individual case.

However, most of the articles cited by us are based on an earlier division, therefore we will refer to the division of diabetes into three groups in the following.

There are different ways to treat the diabetes. Several types of hypoglycemic drugs, which exert antidiabetic effects through various mechanisms, are currently used to treat diabetes mellitus [3]. Metformin, which is example of biguanides, is the first choice drug for type 2 diabetes. This drug is characterized by high effectiveness, low cost, and safety. Treatment of non-insulin-dependent diabetes mellitus (NIDDM) due to insulin dysfunction involves inhibiting or delaying intestinal carbohydrate digestion. Sugars are the main ingredient in our daily diet. One possible therapeutic approach is inhibition of α-glucosidase activity (EC 3.2.1.20). An example of this antidiabetic medicine is acarbose and miglitol, widely used in some parts of Asia [4]. Alfa-glucosidase is located in the epithelium of the small intestine and catalyzes the cleavage of disaccharides and oligosaccharides into glucose. Administration of α-glucosidase inhibitor may prevent digestion of carbohydrates and thus inhibit the rise of blood glucose after meals [5]. In addition, acarbose reversibly inhibits pancreatic α-amylase [4,6].

In turn, metformin increases the insulin sensitivity of tissues and reduces the production of glucose by the liver. However, with prolonged use of this medicine, there is a risk of lactic acidosis development [4,7]. In the case of contraindications to the use of metformin, thiazolidinediones (TZD) such as pioglitazone and rosiglitazone are used, which have many side effects unfortunately. Weight gain and heart failure may be due to the increased fluid retention seen in patients treated with TZD [8]. Sulfonylureas, such as glimepiride and glyburide, are also used to treat diabetes. They work by reducing blood sugar, mainly by increasing insulin release from the Langerhans islets. Their side effect is weight gain. Because of the rather high risk of hypoglycaemia, sulfonylureas are not recommended as the first choice in the pharmacotherapy of type 2 diabetes. In addition, they lose their effectiveness after six years of use in approximately 44% of patients [9]. New antidiabetic drugs include inhibitors of dipeptidyl peptidase 4 (DPP-4, CD26, EC 3.4.14.4), which inhibit the breakdown of intestinal incretin hormones, which increases their bioavailability and duration of action. Examples of drugs in this group approved by the FDA and EMA are sitagliptin, vildagliptin, saxagliptin, linagliptin, and alogliptin. They increase insulin secretion and inhibit glucagon secretion. Nasopharyngitis is the most common side effect caused by DPP-4 inhibitors. Quincke edema and peeling of the skin have also been observed. In addition, severe arthritis and acute pancreatitis have been reported. Incretin therapies include, in addition to DPP-4 inhibitors, also subcutaneously injectable glucagon-like peptide 1 (GLP-1) receptor agonists (e.g., exenatide, liraglutide, lixisenatide) [10]. GLP-1 agonists are parenteral agents that mimic actions of endogenous glucagon-like peptide-1. They lower plasma glucose levels by several mechanisms, including inhibition of glucagon secretion and enhancement of insulin release in a glucose-dependent manner [11]. The last group of antidiabetic drugs are sodium-glucose cotransporter 2 (SGLT 2) inhibitors such as canagliflozin, dapagliflozin, and empagliflozin. Some of the most common side effects of SGLT 2 inhibitors is urogenital tract infection, genital infection, or even breast and bladder cancer [4].

Due to a number of limitations related to the use of existing synthetic antidiabetic drugs, it is currently looking for natural solutions. One of them could be using medicines based on fungi of the genus *Ganoderma.* About 300 species of the genus *Ganoderma* have been identified, most of them naturally occur in tropical regions [12]. Mushrooms belonging to this family are generally not mentioned among the edible, because their fruiting bodies are characterized by corkiness, thickness, high hardness and lack of fleshy texture [13].

Fungi belonging to the *Ganoderma* species have found wide application in natural medicine, especially in traditional Chinese medicine. They are used not only for diabetes but also in the treatment of other chronic diseases such as nephritis, hypertension, arthritis, insomnia, and asthma but also have anti-cancer, anti-hepatotoxic, and immunomodulatory effects [14,15,16]. *G. lucidum* and *G. sinense* hubs are particularly popular although other species, such as *G. capense*, *G. cochlear*, and G. *tsuage* are also used in treatment. The most thoroughly studied species of the genus *Ganoderma* is *G. lucidum*, which is called “Ling-Zhi” in Chinese, “Reishi” in Japanese, and “Yeongji” in Korean. Food products with the addition of this mushroom are described as increasing vitality and longevity [17]. Extracts as well as individual chemical compounds are used to prepare medicines [18,19,20,21].

Analyzing the published results related to the use of substances and extracts derived from fungi of the genus *Ganoderma* as anti-diabetic substances, it can be concluded that two groups of compounds are most important: polysaccharides and terpenoids, therefore their antidiabetic activity will be discussed in this work in the following chapters.

## 2. Hypoglicemic Activity of *Ganoderma* Extracts

The aqueous and alcohol extracts of *G. lucidum* were tested in mice and rats with induced diabetes for lowering blood sugar levels. (Table 1) In research conducted by Seto et al. [22] normal and obese diabetic mice were used. Prior to initiation of plasma, sugar levels measured in plasma were 168.5 mg/dL for normal mice and 668.5 mg/dL for obese mice. A water extract of capsules containing 95% powdered sporocarps of *G. lucidum* and 5% dextrin was used for the tests. After four weeks of administration of the *G. lucidum* extract at a dose of 0.3g/kg, plasma glucose decreased to 68.5 mg/dL in normal mice and 288.4 mg/dL in obese mice.

In turn, in research conducted by Ratnaningtyas et al. [23] alcoholic extract of *G. lucidum* was used, which was administered for 14 days to rats with diabetes artificially induced by Alloxan. Blood glucose levels were determined during tests. At an extract dose of 1000 mg/kg, the glucose level decreased from 384.25 mg/dL to 140.50 mg/dL.

In subsequent studies of hypoglycemic activity normal rats and rats with streptozotocin-induced diabetes were used. During the four-week tests, the serum glucose level was checked. The baseline glucose level in rats without diabetes was 90 mg/dL, whereas in rats with induced diabetes it was 200 mg/dL. Administration of an aqueous extract of *G. lucidum* in an amount of 100 mg/kg reduced glucose levels in normal rats to 60 mg/dL, and in diabetes rats to 150 mg/dL. Increasing the extract dose to 200 mg/kg allowed lowering glucose levels to 45 mg/dL and 90 mg/dL, respectively [24]. In research conducted by Sarker et al. [25] two different extracts were obtained, when dried fruit bodies of *G. lucidum* were extracted with methanol or petroleum ether. Rats that had a plasma glucose level higher than 12 mmol/L were used for the tests. After seven days of administration of the extract, glucose levels were measured. After a further seven-day break, the rats tested were again induced diabetes with dexamethasone. These rats were given *G. lucidum* extracts for the next seven days and plasma glucose levels were determined. The best effects were obtained after using both extracts at a dose of 800 mg/kg. The methanol extract reduced plasma glucose by 36.01% and the ether extract by 55.57% in rats with Alloxan-induced diabetes. In rats with dexamethasone-induced diabetes, glucose levels were reduced by 32.02% (methanol extract) and 51.41% (ether extract).

In subsequent studies, streptozotocin-induced diabetes in rats was given water-alcoholic extract of *G. lucidum* (80%: 20%) at 1 mL/kg for 30 days. After this time, blood sugar levels dropped from 456 mg/dL to 265 mg/dL [26].

## 3. Polysaccharides Isolated from *Ganoderma* Species

Polysaccharides are composed of long chains of monosaccharide units linked together by glycosidic bonds, from which, after hydrolysis, monosaccharides or oligosaccharides are formed. They have a linear to highly branched structure. Polysaccharides have the general formula C_X_(H_2_O)_Y_, where x and y is usually a large number between 200 and 2500. Scientists have found that polysaccharides and glycoconjugates are not only used as energy resources and constituent materials in living organisms, but more importantly, they exist in all structures of the cell membrane and show severe physiological activity [1]. Table 2 summarises enzymes that are directly or indirectly related to diabetes and whose activity is affected by *Ganoderma* polysaccharides.

### 3.1. Polysaccharides Isolated from G. lucidum

Polysaccharides constitute 10–50% of the dry matter of *G. lucidum* fruiting bodies. The content of polysaccharides is different and depends on the growth substrate [39]. Over 200 different polysaccharides were isolated from *G. lucidum* spores, fruiting bodies and mycelium, including *β*-d-glucans, α-d-glucans, α-d-mannans, and polysaccharide-protein complexes [27,40]. The biological effect of glucans depends on their size, degree of branching, water solubility, number of side branches, and length of side chains.

Polysaccharides extracted from hot fruiting body *G. lucidum* (*Gl*-PS) consists of rhamnose, xylose, fructose, galactose, mannose, and glucose with molar ratios of 0.793: 0.964: 2.944: 0.167: 0.384: 7.94, respectively, and are linked together by β-glycosidic linkages. It was a hazel-colored and water-soluble powder [28,29,30,41,42]. Zhang and Lin investigated the hypoglycemic effect of *G. lucidum* polysaccharides using intraperitoneal injection with single doses of 25, 50, and 100 mg/kg of aqueous *G. lucidum* extract in fasting mice. A reduction in serum glucose levels was observed three hours and six hours after extract dosing in a dose-dependent manner. *Gl*-PS at a dose of 100 mg/kg raised circulating insulin levels as early as 1 h after administration by facilitating the influx of Ca^2+^ into pancreatic β cells [1,28,43]. In 2012, another polysaccharide was isolated from *G. lucidum*, which consists of glucose, mannose, xylose, arabinose, galactose, and ribose with a ratio of 68.45:15.87:4.71:4.08:3.78:3.11, respectively [30,44]. Xiao studied the effect of *G. lucidum* polysaccharides at 50 and 100 mg/kg/day administered for seven days to mice with streptozotocin-induced diabetes. Fasting serum glucose concentration, insulin, and epididymal white adipose tissue decreased significantly. In the liver levels of mRNAs such enzyme as glycogen phosphorylase (EC 2.4.1.1), fructose-1,6-bisphosphatase (EC 3.1.3.11), phosphoenolpyruvate carboxykinase (EC 4.1.1.49), and glucose-6-phosphatase (EC 3.1.3.9, G6Pase) were also much lower [27].

In turn, Seto et al. [22] studied the effect of *G. lucidum* extracts administered with feed at 0.03 and 0.3 g per kg of body weight of mice for four weeks. The extract significantly reduced the levels of phosphoenol-pyruvate carboxykinase, which are usually high in diabetic mice [22,28]. *Gl*-PS also inhibits glycogen synthase (EC 2.4.1.11) activity, thereby reducing glucose production in the liver and preventing hyperglycemia [28].

*Gl*-PS protects against alloxane-induced pancreatic islet damage in vitro and in vivo due to free radical capture and inhibition of NF-κB activity [30]. Researchers concluded that low molecular weight polysaccharides have a hypoglycemic effect by regulating the metabolism of anti-apoptosis protein (Bcl-2) and PDX-1 expression, and inhibiting mRNA expression of Bax, iNOS, and Casp-3. In this way, they protect the body’s pancreas cells against apoptosis, in addition, they initiate also their regeneration [31,32].

In 2017, next β-heteropolysaccharide: F31 was separated from *Gl*-PS, with the weight-average molecular weight of 15.9 kDa [33]. The possible mechanism of action of F31 may be associated with the down-regulation of the hepatic glucose regulating enzyme mRNA levels via AMP-activated protein kinase (EC 2.7.11.31, AMPK) activation, improvement of insulin resistance, and a decrease in the epididymal fat/BW ratio [1,33,34].

In turn, LZ-8 may play an important role in type 2 diabetes, reducing lymphocyte infiltration and increasing insulin detection by insulin in beta cells and lowering plasma glucose in non-obese diabetic mice. It has been generally stated that LZ-8 can control diabetes through its immunomodulatory effect [32].

In 1985, Hikino et al. [45] isolated two peptidoglicans with hypoglycemic activity from *G. lucidum*, Ganoderan A (23 kDa), and Ganoderan B (7.4 kDa). These compounds are produced by both mycelium and sporocarps of this mushroom [1,45]. They reported the hypoglycemic effect of ganoderan B, tested on alloxan-induced diabetes mice. This compound has also been reported to increase plasma insulin levels in healthy and glucose-bearing mice. In addition, it showed a significant increase in hepatic glucokinase (GCK, EC 2.7.1.2), phosphofructokinase (PFK-1, EC 2.7.1.11), and glucose-6-phosphate dehydrogenase (G6PDH, EC 1.1.1.49) activity, and also reduced hepatic glucose-6-phosphatase and glycogen synthase activity, thereby reducing hepatic glycogen content [35]. In 1986, a third compound of the same type was isolated, ganoderan C (5.8 kDa), which also had hypoglycemic activity in type 1 diabetic mice [28,46].

Another macromolecular proteoglycan, FYGL, was obtained by extraction with ethanol followed by water and additional purification using Sephadex G75 column chromatography [36]. FYGL with a molecular mass of 72.9 kDa consisted of arabinose, galactose, rhamnose, and glucose in a molecular ratio of 0.08:0.21:0.24:0.47, respectively [30]. It inhibited the action of protein tyrosine phosphatase 1B (PTP1B) in skeletal muscle cells and increased blood insulin levels in a dose-dependent manner. The scientist concluded that this compound showed anti-diabetic and hypolipidemic properties [27,36].

When discussing diabetes, it is worth mentioning the problem of wound healing, which is a frequent serious complication of diabetes. The basic mechanism of cell repair is slowed many times in the disease, which increases the risk of amputation of damaged limbs. In 2012, scientists conducted numerous studies on whether polysaccharides contained in *G. lucidum* can stimulate and accelerate wound healing. Experiments conducted on mice with STZ-induced diabetes showed that the addition of polysaccharides increased the rate of scarring by about 21%. In addition, *G. lucidum* polysaccharides activated wound angiogenesis in part by reducing the oxidative stress of mitochondria by inhibiting the activity and nitration of manganese superoxide dismutase (MnSOD), inhibiting glutathione peroxidase activity (GPx), and reducing phosphorylation of redox enzyme p66Shc [29].

Little data is available on the possible synergistic effects of *G. lucidum* polysaccharides. Recently research [47] was carried out on rats with type 2 diabetes induced by HFD / STZ. Oral administration of a combination of *G. lucidum* inulin and polysaccharides significantly improved body weight and normalized food intake by increasing carbohydrate utilization. This was due to an increase in insulin sensitivity. A reduction in glycogen content was also observed in diabetic rats, which is important because the amount of glycogen in the liver and skeletal muscle is an indirect reflection of insulin activity. The results obtained suggest that the combination of inulin with *G. lucidum* polysaccharides may regulate glucose levels by increasing glycogenesis [47].

The PI3K/Akt pathway is one of the insulin signaling pathways. Phosphoinositide 3-kinase (PI3K), a kinase class, plays an important role in the metabolic action of insulin. Therefore, PI3K dysfunction may affect glucose and lipid metabolism. Protein kinase B (Akt) is a directly acting PI3K molecule that mediates various biological activities of insulin. On the one hand, phosphorylated Akt can increase glucose uptake into cells, facilitating glucose transporter type 4 (GLUT4) translocation to the plasma membrane. On the other hand, it can also effectively modulate glycogen synthesis, reducing glycogen synthase kinase 3β (GSK3β) phosphorylation. The combination of *G. lucidum* polysaccharides with inulin increased gene expression and synthesis of proteins of the PI3K/Akt pathway and increased Akt phosphorylation in diabetic rats compared to the inulin group [47].

Although studies in animal models show the high anti-diabetic potential of *G. lucidum* polysaccharides, there are few clinical data on the use of such compounds in medicines. In 2004, a clinical study of Ganopoly was conducted, which included polysaccharide fractions extracted from *G. lucidum*. It was given to 71 adults with confirmed type 2 diabetes. They received 1800 mg of Ganopoly orally three times a day for 12 weeks. Glycosylated hemoglobin (HbA1C) was analyzed and plasma glucose levels were significantly reduced after 12 weeks of taking Ganopoly [27,48].

### 3.2. Polysaccharides Isolated from G. atrum

PSG-1 was composed of glucose (Glc), mannose (Man), galactose (Gal), and galacturonic acid (GalA) in molar ratio of 4.91:1:1.28:0.71. This heteropolysaccharide comprised a backbone of 1,3-linked and 1,6-linked β-Glc*p* residues substituted at O-3 and O-6 position as the branch points. Side chains were terminated by β-Glc*p*, with the composition of α-1,4-Gal*p*, α-1,4-Gal*p*A, β-1,3-Glc*pi*, and β-1,6-Glc*p*. [30,49] Its hypoglycemic and hypolipidemic effects have been studied in rats with streptozotocin-induced type 2 (STZ) diabetes. PSG-1 administered orally to rats for four weeks at a dose of 200 or 400 mg/kg body weight significantly reduced fasting glucose concentration and serum level of insulin [37,38]. In addition, researchers found that PSG-1 had a protective effect on pancreatic cells by inhibiting the expression of proapoptotic Bax protein and increasing the expression of the antiapoptotic Bcl-2 protein [37].

Cardiovascular complications are the major causes of mortality and morbidity in patients with diabetes. PSG-1 significantly reduced serum total cholesterol, triglyceride, LDL levels, and increased insulin sensitivity and HDL levels, which is important because dyslipidemia is a strong risk factor for the development of cardiovascular disease in patients with diabetes [37]. PSG-1 also improved endothelium-dependent aorta relaxation, increased levels of phosphoinositide 3-kinase (PI3K), phospho-Akt (p-Akt), endothelial nitric oxide synthase (EC 1.14.13.39, eNOS), and level of nitric oxide in diabetic rat aorta [38].

## 4. Terpenoids Isolated from *Ganoderma* Species

Terpenes belong to the largest class of secondary metabolites with diverse biological properties. They are made of isoprene units connected to each other in various ways. Terpenes themselves are hydrocarbons. However, those containing functional groups are called terpenoids. Depending on the number of isoprene units, we distinguish monoterpenes, sesquiterpenes, diterpenes, sesterpenes, triterpenes, tetraterpenes, and politerpenes [50]. Previous studies of the antidiabetic properties of triterpenoids have been associated with inhibition of such enzymes as aldose reductase, α-glucosidase, and HMG-CoA reductase (HMGR) (Table 3).

### 4.1. Triterpenoids Isolated from Ganoderma Species

Previous work indicates that over 140 triterpenoids have been isolated from fungi of the genus *Ganoderma*. Interestingly, as many as 15 triterpenoids have been identified in one species, *G. lucidum* [32]. Triterpenes contain six isoprene units in their structure. They belong to the main class of secondary metabolites. They come from the squalene biosynthesis pathway. Striterpenoids are structurally reminiscent of steroid hormones, so it is speculated that their mechanism of action is associated with glucocorticoid-responsive elements (GRE). Binding to GRE suppresses pro-inflammatory proteins and increases the expression of anti-inflammatory proteins. Because triterpenes are lipophilic in nature, they can bind to cell membranes, thereby affecting their fluidity, which in turn may limit their bioavailability. However, it has been experimentally found that triterpenoids penetrate both cell membranes and the blood–brain barrier and accumulate in the liver, circulatory system, and other tissues [59].

In addition, triterpenoids act as inhibitors of aldose reductase and α-glucosidase, which are actively involved in glucose metabolism [32].

**Figure 1:**
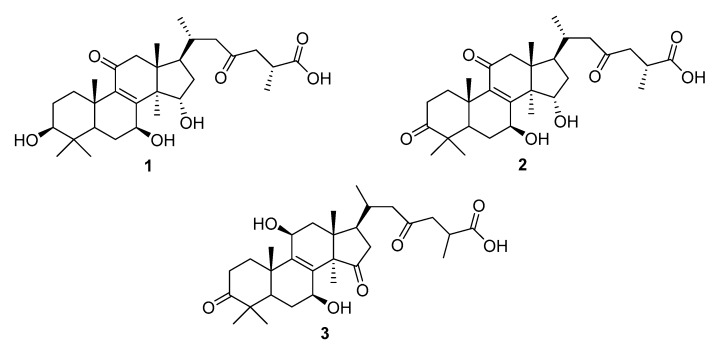
Triterpenes from *G. lucidum.*

Aldose reductase (EC 1.1.1.21) is the first enzyme in the polyol glass, which converts glucose to sorbitol. Accumulation of sorbitol in the body leads to diabetic complications such as neuropathy, nephropathy, cataracts, and retinopathy [60,61]. It was found that the methanol extracts of *G. lucidum* have the highest inhibitory effect on aldose reductase in the group of 17 other edible fungi used therapeutically [51]. It was also found that the *G. lucidum* ethanol extract reduced the level of galactocitol in 50% of galactose-fed rats. In turn, three compounds were identified in the chloroform extract: ganoderic acid C2 (**1**), ganoderenic acid A (**2**), and ganoderic acid Df (**3**), which inhibited this enzyme. Of the above compounds, ganoderic acid Df (**3**) showed the highest activity (22.8 µM) (Figure 1).

The researchers pointed out that this compound (**3**) differs from ganoderenic acid A (**2**) only by the presence of the hydroxyl group at C-11. On this basis, researchers postulate that it must play a key role in the mechanism of inhibition of aldose reductase by ganoderic acid Df, as well as the replacement of the hydroxyl group at C-15 in (**1**) with the carbonyl group in (**3**). An important group is also the hydroxyl group at C-7, which is present in the structure of all three terpenoids mentioned [3,16,32].

**Figure 2:**
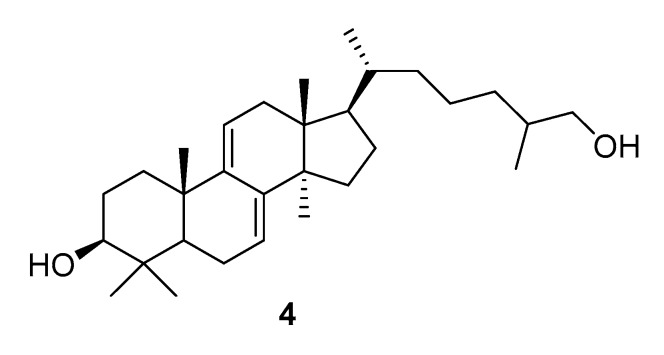
The structure of genoderol B (**4**).

Alpha-glucosidase (EC 3.2.1.20) located in the small intestine epithelium converts disaccharides and oligosaccharides into glucose. For this reason, inhibiting the activity of this enzyme is a common strategy used to reduce hyperglycemia. Among triterpenoid identified in the chloroform extract of *G. lucidum* ganoderol B (**4**) (Figure 2, 119.8 mM) showed greater activity than acarbose commonly used to lower blood glucose levels after a meal [52]. Researchers postulate that the presence of hydroxyl groups at C-3 and at C-25 and a double bond in the side chain are responsible for the high activity of this compound as an α-glucosidase inhibitor [53].

Ethanol extracts of *G. lingzhi* fruit bodies at various stages of development, which contained as many as 25 triterpenes, were also tested for inhibitory activity against α-glycosidase. It was shown that the sample from fruiting bodies collected between 31–34 weeks had the strongest inhibitory properties. The IC_50_ value for this sample was 27 ± 1.4 μg/mL. For comparison, the IC_50_ value for acarbose, a clinically approved inhibitor, was IC_50_ 347 ± 53.3 μg/mL. In general, *G. lingzhi* ethanol extracts at each developmental stage showed a stronger inhibitory effect than the standard inhibitor used [54,55].

**Figure 3:**
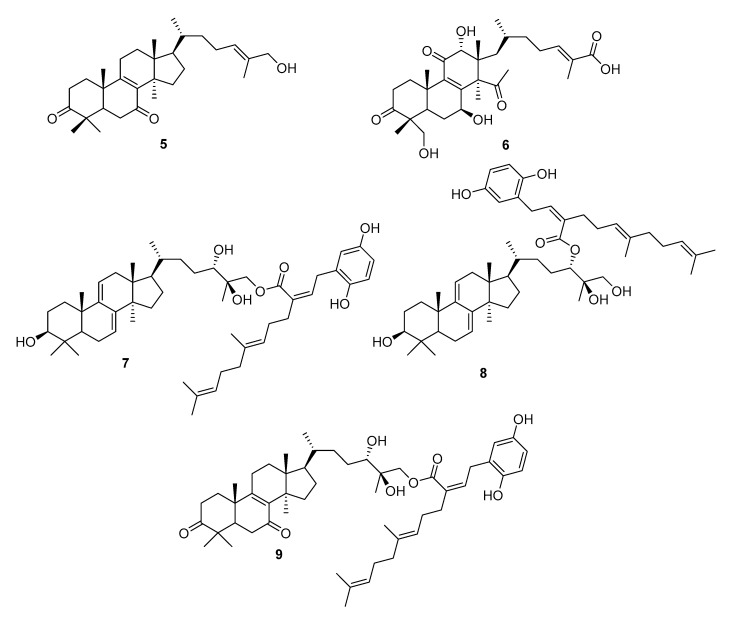
The structures of trirepenoids showed strong inhibitory activity against α-glucosidase.

Also triterpenes isolated from the fruiting bodies of *Ganoderma leucocontextum* and *G. lucidum* have strong inhibitory properties for diabetes-related enzymes. Ganoderone A (**5**) and ganoleuconins E (**6**), M (**7**), N (**8**), and P (**9**) (Figure 3) showed strong inhibitors activity against α-glucosidase from yeast with IC_50_ values of 12.7, 13.6, 2.5, 5.9, and 13.7 μM, respectively [56].

Three compounds are shown as potential inhibitors of activity against α-glucosidase from rats. In vitro, the following IC_50_ values were obtained for ganoleuconin M (**7**)—13.6 ± 3.1 μM; ganoleuconin N (**8**)—2.5 ± 0.7 μM; and ganoleuconin P (**9**)—5.9 ± 2.2 Μm [56] (Figure 3).

**Figure 4:**
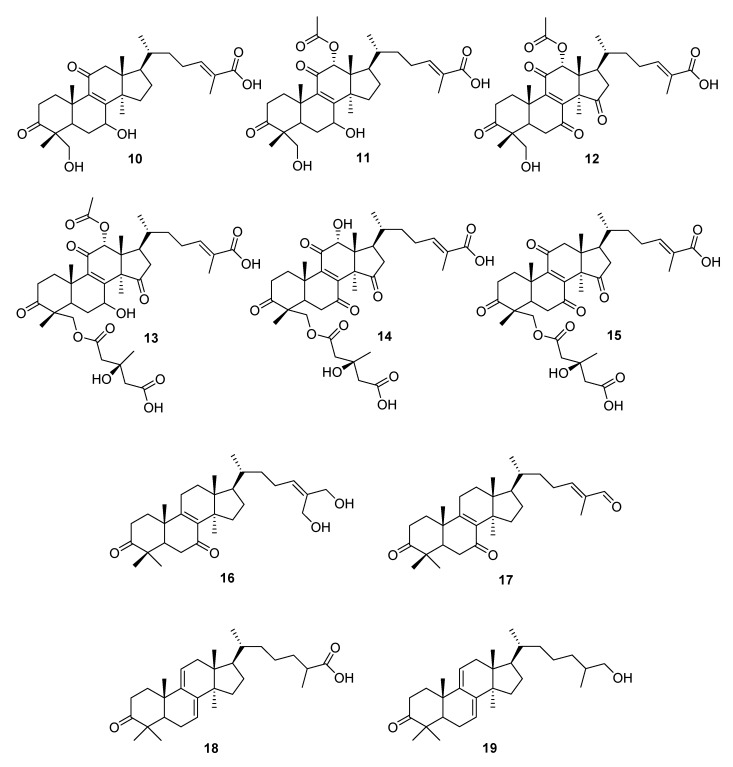
The triterpenoids isolated from *G. leucocontextum* and *G. lucidum* showed strong inhibitory activity against HMG-CoA reductase.

In turn, ganoleuconins A (**10**), C (**11**), F (**12**), J (**13**), K (**14**), L (**15**), M (**7**) N (**8**), ganoderiol J (**16**), ganoderic acid DM (**17**), ganoderic acid S (**18**), and ganoderic acid Y (**19**) (Figure 4) showed much stronger inhibitors activity against HMG-CoA reductase (EC 1.1.1.34) than the positive control atorvastatin. The IC_50_ values of these compounds were in the range of 8–00 μM [56].

### 4.2. Meroterpenoids Isolated from Ganoderma Species

A special case of terpenoid compounds are meroterpenes, which are a combination of terpenoid and phenolic compounds. Aromatic meroterpenoids formed partly from mevalon acid and biogenetic shikimic acid pathways [18,50]. The characteristic structure of meroterpenoids indicates the possible biological activities of newly detectable compounds. This is extremely important especially when creating new drugs.

**Figure 5:**
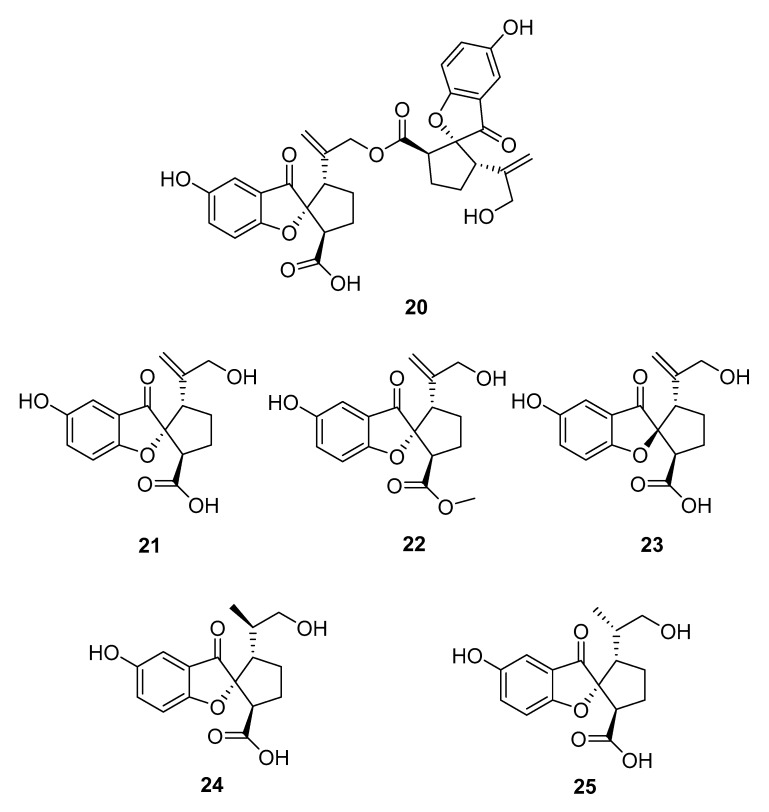
The meroterpenoids from *Ganoderma leucocontextum.*

Meroterpenes from fungi of the genus *Ganoderma* (GMs) contain in their structure a polyunsaturated terpenoid part and 1,2,4-trisubstituted phenyl [18]. This structure is probably related to the degradation of lignin to the phenyl group by the liginolytic enzymes of *Ganoderma* while the terpenoid parts were further assembled under prenyltransferase. Wang [58] presented the results of isolated molecules from *G. leucocontextum*. These were the compounds ganoleucin D (**20**), spiroapplanatumine K (**21**), spiroapplanatumine L (**22**), (±)-spirolingzhine A (**23**, **24**), and spirolingzhine D (**25**). (Figure 5)

Compounds **20**, **22**–**25** have been shown to inhibit aldose reduction from bovine lens tissue with IC_50_ values in the range 9.4−28.9 μM in in vitro testing. In addition, for the strongest inhibitor, spirolingzyn D (**25**), an in silico docking study was performed with bovine lens aldose reductase, to confirm the interaction with the DDI-CPI tool, a web-based server that can predict drug–drug interactions via the chemical–protein interactome. The molecular docking results showed that the binding pocket involves amino acid residues Trp20, Ser22, Pro23, Val47, Tyr48, Gln49, Asn50, and Pro218. Theoretical tests have been confirmed by laboratory tests which indicated compound 25 shows the strongest inhibitors effect with an IC_50_ value of 9.4 μM. In addition, the tested compounds were tested for their inhibitory activity on HMG-CoA reductases. The studies presented showed that only compound **20** showed a moderate inhibitory effect on HMG-CoA reductases, with an IC_50_ of 27.9 μM [57].

Eight other meroterpenes also isolated from the fruiting bodies of *G. leucocontextum* were studied for the inhibitors effects on HMG-CoA reductase and α-glucosidase. Ganomycin I (**26**), (**27**), and (**28**) showed stronger inhibitors activity against HMG-CoA reductase than the standards. The positive control in this study was atorvastatin.

**Figure 6:**
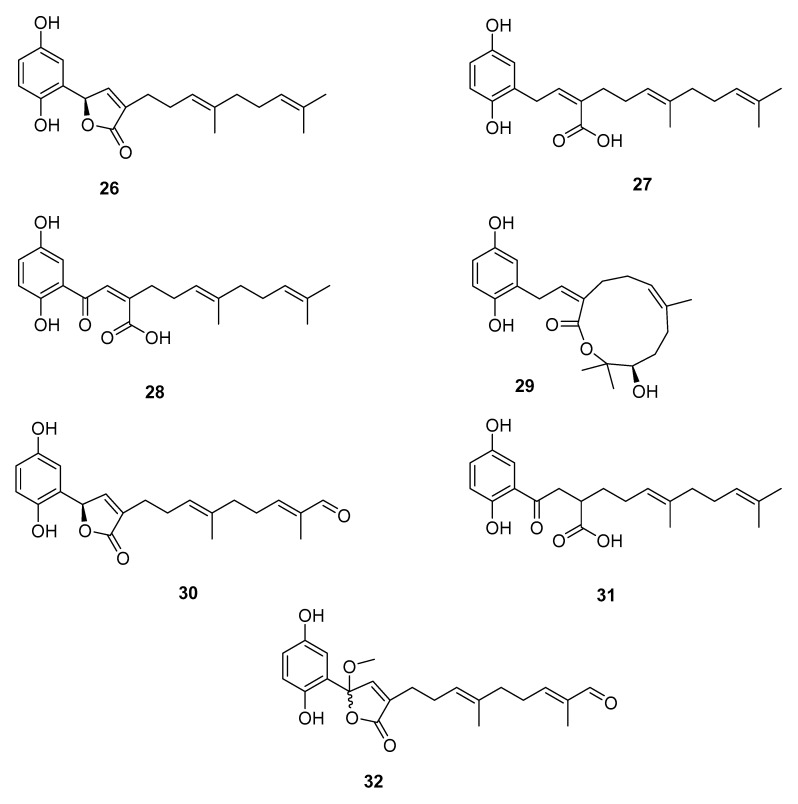
The meroterpenes from *Ganoderma leucocontextum*.

The meroterpenes (Figure 6) presented potent noncompetitive inhibitors activity against α-glucosidase from both yeast and rat small intestinal mucosa. Ganomycin I (**26**) was the most potent inhibitor against both α-glucosidase and HMG-CoA reductase. Pharmacological results showed that ganomycin I (**26**) exerted potent and efficacious hypoglycemic, hypolipidemic, and insulin-sensitizing effects in KK-Ay mice [58].

## 5. Summarized Treatment Mechanism by *Ganoderma*

**Scheme 1:**
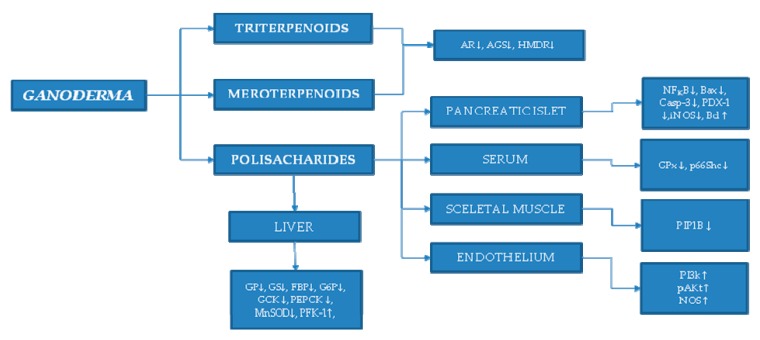
The antidiabetes activity of *Ganoderma*.

Diabetes mellitus is a multifactorial metabolic disorder and is generally caused by the impairment or insufficiency of β-cells in the pancreas that diminishes insulin biosynthesis and gradually deteriorates whole body functions. In contrast to physiological glucose concentration, these glucose levels negatively affect a greater number of organs and tissues. Due to chronic hyperglycemia, decreasing insulin secretion, as well as up-surging insulin resistance, provides glucose toxicity. Hyperglycemia produces an elevated reactive oxygen species and reactive nitrogen species in β-cells, providing succeeding impairment to cellular mechanisms. Glucotoxicity therefore decreases the antioxidant defence system in the body [62]. Polysaccharides of *G. lucidum* significantly down-regulated such enzymes as iNOS, p66Shc, manganese superoxide dismutase, nitric oxide synthase, and glutathione peroxidase (Scheme 1, Table 2).

The next dangerous process observed during diabetes mellitus is apoprosis of β-cells. Though the underlying mechanism of β-cell apoptosis in T2DM is complex and debated [63] the prevention of β-cell apoptosis and connected elements are a vital approach for treating T2DM. Polysaccharide PSG-1 from *G. atrum* administration in diabetic animals significantly reduced fasting blood glucose, plasma insulin, and expression of Bax and improved expression of Bcl2 in the high-fat diet STZ-induced diabetic rats. Also polysaccharides from *G. lucidum* significantly up-regulated Bcl-2 and down-regulated Bax and caspase 3 in the pancreatic cells compared to those of STZ diabetic animals. The results strongly suggested that polysaccharide from *G. lucidum* exerted a hypoglycemic potential by inhibiting the β-cell apoptosis in diabetic rats. Peptidoglicans of *G. lucidum* ganoderan A, and ganoderan B promote insulin release, while allowing Ca^2+^ to flow into islet β cells. In turn, the mechanism of action of β-heteropolysaccharide F31 may be related to the down-regulation of AMPK-activated hepatic glucose regulator mRNA, the improvement of insulin resistance, and the decrease in epididymal fat/body weight ratio [1].

Hypoglycemic activity of *G. lucidum* polysaccharides is also connected with regulating the expression of several key enzymes in the hepatic glucose metabolism pathway, such as glucokinase, glucose-6-phosphatase, glucose-6-phosphate dehydrogenase, phosphoenolpyruvate carboxykinase, fructose-1,6-bisphosphatase, phosphofructokinase, and glycogen synthase [32].

Dietary carbohydrates are naturally digested into monosaccharides, such as glucose and fructose; these monosaccharides can be readily uptaken by the small intestine and transfer into the blood circulation. Assimilation of oligosaccharides is connected with activity of α-glucosidase in the small intestine [62]. This enzyme is effectively inhibited by several triterpenoids of *Genoderma* (Table 3).

Due to the satisfactory effect and medical potential of *Ganoderma*, they were used in clinical studies for various indications, not only diabetes [6,7,8,9,10,11,12,13,14,15,16,17,18,19,20,21,22,23,24,25,26,27,28,29,30,31,32,33,34,35,36,37,38,39,40,41,42,43,44,45,46,47,48,49,50,51,52,53,54,55,56,57,58,59,60,61,62,63,64,65,66].

## 6. Future Prospects

Numerous studies on the broad spectrum of biological activity of mushrooms used in traditional Chinese medicine have been concluded. *G. lucidum* is one of the famous medicinal mushrooms especially in Asia countries. A number of pharmacological studies carried out on this mushroom have proved its efficacy and therapeutic potential. Moreover, various studies emphasized that it has few or no side effects, which confirms the safety profile of this mushroom.

Although great progress has been made in the research on *Ganoderma* species polysaccharides and triterpenoids with antidiabetic activity in recent years [67,68], the research has large gaps before its practical application as medicines. There are several areas for future research:

- standardization of *Ganoderma* species sources;
- the relationship between the structure and function of *Ganoderma* species individual polysaccharides and triterpenoids is not yet fully clear;
- investigation of the possible synergistic or antagonistic effects of active substances with food ingredients;
- developing more efficient and economic approaches for the preparation and modification of the most active compounds;
- polysaccharides structure determination methods are far from reaching the protein and nucleic acid structure determination as automated, micro-quantified, and standardized;
- polysaccharides in vivo mechanism of action is not yet fully clear, especially lacking in full toxicological profile.

Based on statements above, the conclusion is that, although several preclinical and clinical studies of polysaccharides and triterpenoids of *Ganoderma* species have been conducted to search for miraculous antidiabetic properties, still more clinical studies are needed to endorse their safety and efficacy.

## Figures and Tables

**Table 1:** Research activity of extracts of *Ganoderma* in animal models of diabetes.

Source	Extract	Experimental Model	Dose	Concentration of Glucose	Reference
*G. lucidum*	Water extract of powder of the capsules of *G. lucidum* contains 95% *Ganoderma* extract (obtained from the whole fruit body) and 5% dextrin.	C57BL/KsJ mice (female; 6 months old) (normal mice)	0 g/kg	168.5 mg/dL	[22]
			0.003 g/kg	161.6 mg/dL	
			0.03 g/kg	126.5 mg/dL	
			0.3 g/kg	68.5 mg/dL	
		C57BL/KsJ mice (female; 6 months old) (diabetic mice)	0 g/kg	668.5 mg/dL	
			0.003 g/kg	645.9 mg/dL	
			0.03 g/kg	441.5 mg/dL	
			0.3 g/kg	288,4 mg/dL	
*G. lucidum*	Ethanol extract of powdered fruiting bodies	white rats (*Rattus norvegicus)* derived from Wistar line, male, 2–3 weeks old	0 mg/kg	435.75 mg/dL	[23]
			250 mg/kg	312.00 mg/dL	
			0 mg/kg	311.00 mg/dL	
			500 mg/kg	203.50 mg/dL	
			0 mg/kg	384.25 mg/dL	
			1000 mg/kg	140.50 mg/dL	
*G. lucidum*	Water extract of fruiting bodies	male Wistar rats weighing 85 – 100 g, normal rats	0 mg/kg	90 mg/dL	[24]
			100 mg/kg	60 mg/dL	
			200 mg/kg	45 mg/dL	
		male Wistar rats weighing 85 – 100 g, diabetic rats	0 mg/kg	200 mg/dL	
			100 mg/kg	150 mg/dL	
			200 mg/kg	90 mg/dL	
*G. lucidum*	Methanol extract of fruiting bodies	Long-Evans male rats (4 weeks age, weight 83.5 g), induced with alloxan	0 mg/kg	14.5 mg/dL	[25]
			200 mg/kg	12.5 mg/dL	
			400 mg/kg	12 mg/dL	
			600 mg/kg	10 mg/dL	
			800 mg/kg	10 mg/dL	
	Petroleum ether extract of fruiting bodies		0 mg/kg	14.5 mg/dL	
			200 mg/kg	10 mg/dL	
			400 mg/kg	9.5 mg/dL	
			600 mg/kg	7 mg/dL	
			800 mg/kg	7 mg/dL	
	Methanol extract of fruiting bodies	Long-Evans male rats (4 weeks age, weight 83.5 g), induced with corticosteroid	0 mg/kg	17.5 mg/dL	
			200 mg/kg	14 mg/dL	
			400 mg/kg	12.5 mg/dL	
			600 mg/kg	10 mg/dL	
			800 mg/kg	9.5 mg/dL	
	Petroleum ether extract of fruiting bodies		0 mg/kg	17.5 mg/dL	
			200 mg/kg	10 mg/dL	
			400 mg/kg	7.5 mg/dL	
			600 mg/kg	5 mg/dL	
			800 mg/kg	4.8 mg/dL	
*G. lucidum*	Water extract of fruiting bodies (80%) and alcohol extract of fruiting bodies (20%)	Male Wistar rats aged four weeks and weighing 250 to 300 g	0 mL/kg	456 mg/dL	[26]
			1 mL/kg	265 mg/dL	

**Table 2:** Antidiabetic activity of *Ganoderma* polysaccharides.

Polysaccharide	Tissue	Enzyme	Reference
Hot water extract	liver	glycogen phosphorylase (GP) ↓	[27]
		fructose-1,6-bisphosphatase (FBP) ↓	
		glucose-6-phosphatase (G6P) ↓	
		phosphoenolpyruvate carboxykinase (PEPCK) ↓	[27,28]
		glycogen synthase (GS) ↓	[28]
		manganese superoxide dismutase (MnSOD) ↓	[29]
	serum	glutathione peroxidase (GPx) ↓	
		p66Shc ↓	
	pancreatic islet	nuclear factor kappa (NF-κB) ↓	[30]
		insulin promoter factor 1 (PDX-1) ↓	[31,32]
		Bax ↓	
		calcium-insensitive nitric oxide synthase (iNOS) ↓	
		caspase 3 (Casp-3) ↓	
F31	liver	AMP-activated protein kinase (AMPK) ↓	[1,33,34]
ganoderan B	liver	glucokinase (GCK) ↑	[35]
		Phosphofructokinase (PFK-1) ↑	
		glucose-6-phosphate dehydrogenase (G6PDH) ↑	
		glucose-6-phosphatase (G6P) ↓	
		glycogen synthase (GS) ↓	
FYGL	skeletal muscle	tyrosine phosphatase 1B (PTP1B) ↓	[36]
PSG-1 (*G. atrum*)	pancreatic cells	Bax ↓	[37]
		Bcl-2 ↑	
	endothelium	phosphoinositide 3-kinase (PI3K) ↑	[38]
		phospho-Akt (p-Akt) ↑	
		nitric oxide synthase (eNOS) ↑	

**Table 3:** Antidiabetic activity of *Ganoderma* triterpenoids.

Species	Terpenoids	Enzyme	Reference
*G. lucidum*	ganoderic acid C2 (**1**), ganoderenic acid A (**2**) ganoderic acid Df (**3**)	aldose reductase (AR) ↓	[3,16,32,51]
*G. lucidum*	ganoderol B (**4**)	α-glucosidase (AGS) ↓	[52,53]
*G. lingzhi*	3,12,20-trihydroxy-7,11,15-trioxolanost-8,16,24- -trien-26-oic acid,ethanolic extract of *G. lingzhi*		[54,55]
*G. leucocontexum*	ganoderone A (**5**), ganoleuconin E, M, N, P, (**6**–**9**)		[56]
*G. leucocontexum*	ganoleuconin A (**10**), C (**11**), F (**12**), J (**13**), K (**14**),L (**15**), M (**7**), N (**8**), ganoderiol J (**16**)	HMG-CoA reductase (HMGR) ↓	[56]
*G. leucocontextum*	ganoleucin D (**20**), spiroapplanatumine L (**22**) (±)-spirolingzhine A (**23**, **24**) spirolingzhine D (**25**)	aldose reductase ↓	[57]
	ganoleucin D (**20**)	HMG-CoA reductase ↓	
	ganomycin I (**26**)		[58]
